# A Comprehensive Molecular and Serological Investigation of Hepatitis A Virus Among Patients With Suspected Acute Hepatitis: A Brazilian Study

**DOI:** 10.1002/jmv.70449

**Published:** 2025-06-18

**Authors:** Ketty Gleyzer de Oliveira, Mariana Pinheiro Alves Vasconcelos, Julia Teixeira Ton, Silvia Naomi de Oliveira Uehara, Roberta Sitnik, Denize Ornelas Pereira Salvador de Oliveira, Layze Castberg, Ricardo Andreotti Siqueira, Peter James Robinson, Thaís Senna de Paula Domingues, Caroline Thomas Panico, César Augusto Inoue, Maira Marranghello Maluf, Fernanda de Mello Malta, Deyvid Amgarten, Erick Dorlass, Pedro Sebe, Arlene S. Pinto, Cirley M. D. O. Lobato, Adalgisa Ferreira, Elodie Hyppolito, Raymundo Paraná, Maria Isabel Schinoni, Edmundo Pessoa de Almeida Lopes, Magali C. Luiz, Raquel F. L. Garcia, Dennis Armando Bertolini, Mário Reis Álvares‐da‐Silva, Raul Salinas Arrojo, Eduardo Emerim, Gabriela P. Coral, Paulo R. Acosta, Marcello Lucena, Rosângela Teixeira, Mônica da Costa Guedes, Livia Melo Villar, Lia Lewis Ximenez, Clarice Gdalevici, Maria Cássia Jacintho Mendes‐Correa, Flair José Carrilho, Gerson Sobrinho Salvador de Oliveira, Paulo Roberto A. Ferreira, Maria Lucia Gomes Ferraz, Ana Catharina de Seixas Santos Nastri, Pablo Andres Munoz Torres, Glória Selegatto, Luciana Vilas Boas Casadio, Simone Tenore, Olavo Henrique Munhoz Leite, Fernanda Fernandes Souza, Tania Reuter, Francisco Souto, Michele Nascimento‐Sales, Ana Paula Maciel Gurski, Andréa Salomão, Bruna Emanuelle Alvarenga Fanis, Maria Cristina Pimenta, Elton Carlos de Almeida, Flávia Moreno Alves de Souza, Gerson Fernando Mendes Pereira, Mario Peribañez Gonzalez, Michele Soares Gomes‐Gouvea, Raymundo Soares de Azevedo, João Renato Rebello Pinho

**Affiliations:** ^1^ Laboratório de Patologia Clínica e de Anatomia Patológica, Hospital Israelita Albert Einstein São Paulo Brazil; ^2^ LIM07, Instituto de Medicina Tropical, Departamento de Gastroenterologia, Hospital das Clínicas HCFMUSP, Faculdade de Medicina da Universidade de São Paulo São Paulo Brazil; ^3^ Centro de Pesquisa em Medicina Tropical, Secretaria de Estado da Saúde de Rondônia Porto Velho Rondônia Brazil; ^4^ Hospital Dia Esterina Corsini de Campo Grande, Universidade Federal de Mato Grosso do Sul Campo Grande Mato Grosso do Sul Brazil; ^5^ Fundação de Medicina Tropical Dr. Vieira Machado de Manaus, Secretaria de Estado da Saúde do Amazonas Manaus Amazonas Brazil; ^6^ Unidade de Saúde de Referência de Doença de Rio Branco, Secretaria Municipal da Saúde Rio Branco Acre Brazil; ^7^ Departamento de Medicina Universidade Federal do Maranhão São Luís Maranhão Brazil; ^8^ Hospital São José das Doenças Infecciosas de Fortaleza, Serviço de Transplante de Fígado do Hospital Universitário Walter Cantídio, Universidade Federal do Ceará Fortaleza Ceará Brazil; ^9^ Hospital Universitário Professor Edgar Santos de Salvador, Faculdade de Medicina da Universidade Federal da Bahia Salvador Bahia Brazil; ^10^ Hospital de Clínicas, Universidade Federal de Pernambuco Recife Pernambuco Brazil; ^11^ Hospital Nereu Ramos, Secretaria de Estado da Saúde Florianópolis Santa Catarina Brazil; ^12^ Hospital Municipal São José, Prefeitura de Joinville Santa Catarina Brazil; ^13^ Departamento de Análises Clínicas e Biomedicina Universidade Estadual de Maringá Maringá Paraná Brazil; ^14^ Hospital de Clínicas de Porto Alegre, Universidade Federal do Rio Grande do Sul Porto Alegre Rio Grande do Sul Brazil; ^15^ Centro de Testagem e Aconselhamento Santa Marta de Porto Alegre, Prefeitura de Porto Alegre Rio Grande do Sul Brazil; ^16^ Santa Casa de Porto Alegre, Universidade Federal de Ciências da Saúde de Porto Alegre Porto Alegre Rio Grande do Sul Brazil; ^17^ Consórcio Intermunicipal de Saúde de Pato Branco Pato Branco Paraná Brazil; ^18^ Secretaria Municipal de Saúde de Florianópolis, Prefeitura de Florianópolis Florianópolis Santa Catarina Brazil; ^19^ Equipe de Gastrohepatologia, Hospital Felício Rocho Belo Horizonte Minas Gerais Brazil; ^20^ Especialista em Saúde Pública, Secretaria Municipal de Saúde do Rio de Janeiro Rio de Janeiro Brazil; ^21^ Instituto Oswaldo Cruz, Fundação Oswaldo Cruz Rio de Janeiro Brazil; ^22^ Polo de Hepatites, Instituto de Assistência dos Servidores do Estado do Rio de Janeiro Rio de Janeiro Brazil; ^23^ Departamento de Moléstias Infecciosas e Parasitárias Faculdade de Medicina da Universidade de São Paulo São Paulo Brazil; ^24^ Hospital Universitário de São Paulo, Faculdade de Medicina da Universidade de São Paulo São Paulo Brazil; ^25^ Escola Paulista de Medicina, Universidade Federal de São Paulo São Paulo Brazil; ^26^ Centro de Referência e Tratamento de IST AIDS. Secretaria de Estado da Saúde de São Paulo São Paulo Brazil; ^27^ Disciplina de Infectologia do Centro Universitário Faculdade de Medicina do ABC de São Paulo São Bernardo São Paulo Brazil; ^28^ Hospital das Clínicas de Ribeirão Preto, Faculdade de Medicina de Ribeirão Preto da Universidade de São Paulo São Paulo Brazil; ^29^ Hospital Universitário Cassiano Antônio Moraes, Universidade Federal do Espírito Santo Vitória Espírito Santo Brazil; ^30^ Hospital Universitário Júlio Muller, Ambulatório 3 de Infectologia de Cuiabá Mato Grosso Brazil; ^31^ Professora de Ciências Biológicas e da Saúde, Universidade Cruzeiro do Sul de São Paulo São Paulo Brazil; ^32^ Departamento de HIV/Aids, Tuberculose, Hepatites Virais e Infecções Sexualmente Transmissíveis da Secretaria de Vigilância em Saúde e Ambiente do Ministério da Saúde (Dathi/SVSA/MS) Brasília Brazil; ^33^ Instituto de Infectologia Emílio Ribas de São Paulo, Secretaria de Estado da Saúde São Paulo São Paulo Brazil; ^34^ LIM01, Departamento de Patologia Faculdade de Medicina da Universidade de São Paulo São Paulo Brazil; ^35^ LIM03, Hospital das Clínicas HCFMUSP, Faculdade de Medicina da Universidade de São Paulo São Paulo Brazil

**Keywords:** Brazil, epidemiology, HAV, immunization program, serology, viral hepatitis

## Abstract

Hepatitis A Virus (HAV) infects millions of individuals annually and is a major cause of acute viral hepatitis worldwide. This study aims to (1) assess HAV infection in suspected acute hepatitis patients at public healthcare institutions in Brazil; (2) evaluate the proportion of immunized individuals against HAV; (3) identify HAV genotypes; (4) examine the association between HAV infection and demographic data, as well as exposure to risk factors. This is a prospective, observational multicenter study conducted in primary health services in Brazil from October 2019 to May 2023, involving 1721 patients with suspected acute hepatitis. Acute HAV infection was identified in 108 (6.3%) patients, predominantly in young men (80%) and from South and Southeast regions of Brazil (97%). Anti‐HAV IgG, indicating previous exposure or vaccination, was detected in 78.6% of individuals (74% in the South to 91% in the North). Genotype I.A was found in all cases and approximately 450 mutations were identified, most of them in the structural proteins VP1‐3. Two viral groups were identified and related to two introductions of the virus: cosmopolitan sequences from North America, South America, and Europe, and a minor group of Brazilian sequences similar to Asian and South American ones. The high incidence of acute HAV infections highlights the need for targeted prevention and vaccination strategies. The characterization of HAV genetic diversity and molecular epidemiology contributes to monitoring and identifying emerging outbreaks.

**Abbreviations**
AHA: acute hepatitis A
bp: base pairs
CA: California
CAAE: certificate of ethical presentation
CI: confidence interval
CMIA: chemiluminescent microparticle immunoassay
CONEP: National Research Ethics Council
CRIE: Special Immunobiological Reference Centers
C‐terminal: carboxyl‐terminal end of a protein
DSP: dried sample preparation
ESCRT: endosomal sorting complex required for transport
GTR + G + I: GTR (General Time Reversible)+ G (Gamma Distribution) + I (Proportion of Invariant Sites)
HAV: Hepatitis A Virus
HAVAb: antibodies to Hepatitis A Virus
HSCT: hematopoietic stem cell transplantation (HSCT)
ICF: informed consent form
IgG: immunoglobulin G
IgM: immunoglobulin M
IL: Illinois
IQR: interquartile range
MAFFT: multiple alignment using fast fourier transform
min: minutes
mNGS: metagenomic next generation sequencing
MSM: men who have sex with men
NAFLD: nonalcoholic fatty liver disease
NCBI: National Center for Biotechnology Information
OR: odds ratios
*p*
: *p* value (probability value)
P1: structural protein 1 (HAV)
P2: structural protein 2 (HAV)
PCR: polymerase chain reaction
PDCD6IP: programmed cell death 6‐interacting protein
PWID: people who inject drugs
REDCap: research electronic data capture
RefSeq: Reference Sequence: a comprehensive, integrated, nonredundant set of sequences maintained by NCBI
RNA: ribonucleic acid
RT‐PCR: reverse transcription polymerase chain reaction
s: seconds
SOT: solid organ transplant recipients
ULN: upper limit of normal
USA: United States of America
VP1: viral protein 1
VP2: viral protein 2
VP3: viral protein 3
VP4: viral protein 4
YPX₃L: a specific amino acid motif (Y = Tyrosine, P = Proline, X = Any amino acid, and the subscript 3 indicates three repetitions of X, followed by L = Leucine

## 1 Introduction

Hepatitis A virus (HAV) is a significant cause of acute viral hepatitis worldwide. The virus belongs to the *Picornaviridae* family, *Hepatovirus* genus and has a single open reading frame that encodes all its proteins: P1 region encodes structural proteins (VP4, VP2, VP3 and VP1), while P2 and P3 regions encode nonstructural proteins involved in viral replication. Analyzing HAV genomic sequences helps identify genotypes and subgenotypes, offering insights into viral evolution, genetic diversity, and global spread [1].

Although global seroprevalence of HAV infection is high, it varies based on geographical and demographic factors, and this infection is still pending in hyperendemic regions and is emerging in low‐endemic regions [2]. Annually, HAV is estimated to cause over 150 million clinical cases, but only 1.5 million are reported, indicating significant underestimation, especially among younger age groups with asymptomatic cases. In high‐income countries, HAV accounts for 20%–25% of viral hepatitis cases, while in low‐ and middle‐income countries, the proportion is expected to be higher [3].

The morbidity and mortality rates of HAV infection differ across regions and populations. Globally, the mortality rate associated with HAV remains relatively low, accounting for approximately 0.5% of deaths caused by viral hepatitis [4]. In Brazil, between 2000 and 2023, a total of 171 255 cases of hepatitis A were reported, with the Northeast region accounting for the largest share (29.7%). During 2017 and 2018, incidence rates in São Paulo and Rio de Janeiro states rose to 2.5 and 2.8 cases per 100 000 inhabitants, respectively. The incidence rate of hepatitis A in Brazil increased again, climbing from 0.4 (2019) to 1 (2023) case per 100 000 inhabitants. This surge was predominantly driven by the South and Southeast regions, which reported rates of 2.1 and 1.3 cases per 100 000 inhabitants, respectively. Between 2000 and 2022, 1380 deaths attributed to hepatitis A were recorded, with the majority occurring in individuals aged over 60 years [5].

Patients with pre‐existing chronic liver diseases or compromised immune systems are at higher risk of developing severe forms of hepatitis A. Chronic liver diseases include conditions such as cirrhosis, nonalcoholic fatty liver disease (NAFLD), alcoholic liver disease, autoimmune hepatitis, primary biliary cholangitis, and hepatocellular carcinoma. These conditions weaken the liver's ability to recover from infections, making patients more vulnerable to complications like fulminant hepatitis. Immunocompromised individuals also face heightened risks. This group includes people undergoing chemotherapy, organ or stem cell transplants, or immunosuppressive therapy. It also encompasses those with advanced HIV/AIDS, primary immunodeficiency disorders, and individuals on long‐term high‐dose corticosteroids or other immunosuppressive medications. Additionally, older adults, particularly those over 60 years of age, are more likely to experience severe outcomes, including fulminant hepatitis, which can lead to acute liver failure [4]. In Brazil, this risk is particularly pronounced when individuals from lower socioeconomic status are affected as they may have limited access to medical assistance [6].

HAV vaccination plays a crucial role in preventing infection and controlling outbreaks worldwide. Introduction of vaccines has significantly reduced the incidence of hepatitis A in many countries, especially in regions with poor sanitary conditions and hygiene practices [4]. In Brazil, hepatitis A vaccine is part of the childhood vaccination schedule, with a single dose at 15 months of age (it can be used from 12 months to under 5 years–4 years, 11 months, and 29 days). Additionally, the vaccine is available at the Special Immunobiological Reference Centers (CRIE), in a 2‐dose schedule—with a minimum interval of 6 months—for people over 1 year of age with the following conditions: chronic liver diseases of any etiology, including chronic HBV and/or HCV infection; chronic HBV carriers; coagulopathies; people living with HIV or AIDS; therapeutic or disease‐induced immunosuppression; storage diseases; cystic fibrosis; trisomies; candidates for solid organ transplantation, registered in transplant programs; solid organ transplant recipients (SOT); hematopoietic stem cell transplantation (HSCT); solid organ or hematopoietic stem cell donors registered in transplant programs; hemoglobinopathies; anatomical or functional asplenia and related diseases [7].

The different genotypes and subgenotypes of HAV have a characteristic geographical distribution. Genotype I is prevalent in different parts of the world, and the occurrence of subgenotype A is higher than that of B, with both reported in co‐circulation in some regions, such as South Africa, Brazil, and Israel. The co‐circulation of subgenotypes I.A and III.A can be observed in some countries like India and others in Central Asia, which belonged to the former Soviet Union. Subgenotype I.A is widely distributed worldwide, with strains found circulating in North and South America, Europe, Asia, and Africa. Subgenotype IB is prevalent in Middle Eastern countries and has isolated strains in countries in Europe, North Africa, and South America, as well as in Australia, Japan, and Jordan. In Brazil, this subgenotype has been found in outbreaks, which may suggest its greater dissemination than previously expected. Genotype II has been identified in France and Sierra Leone but is rarely found. Some studies suggest its origin in endemic regions such as West Africa and admit that the reasons for its limitation in the world are unknown. Genotype III is the only one with strains isolated from humans and nonhuman primates. Like genotype I, it has a wide global distribution and is frequently found in European countries such as France and Italy, and in Asian countries where subgenotype III.A seems to be endemic, such as Nepal, India, Malaysia, and Sri Lanka. In North America, this genotype circulates mainly in the United States. This subgenotype was described in cases in North Brazil in the 1980s and due to its wide and growing geographical distribution observed among humans, a human origin for this genotype is suggested [8].

The clinical presentation of hepatitis A virus (HAV) infection can vary depending on the genotype. While most HAV infections are asymptomatic or mild, certain genotypes have been associated with more severe outcomes. For example, genotype I.B has been found more frequently among cases of acute liver failure compared to non‐liver failure cases, suggesting its potential greater virulence [9].

USA Guidelines for HAV provided by the Centers for Disease Control and Prevention (CDC, Atlanta, GA, USA) recommends vaccination to that all children between 12 and 23 months old, children and adolescents 2–18 years old who have not previously been vaccinated, and adults at risk for HAV infection or severe disease from HAV infection; emphasizes the importance of vaccination, good hygiene practices, and safe food and water consumption to prevent HAV transmission; and offers resources for managing HAV outbreaks, including those linked to food sources and person‐to‐person contact [10]

Japan Guidelines for HAV were provided by the National Institute of Infectious Diseases (NIID): strong recommendation for vaccination among travelers to HAV‐endemic areas, medical practitioners, patients with chronic liver diseases, and high‐risk groups such as men who have sex with men (MSM) and people who inject drugs (PWID); HAV as a Category IV infectious disease under the Infectious Diseases Control Law requires notification of all diagnosed cases, including asymptomatic carriers; emphasis on the importance of vaccination and awareness‐raising about HAV as a sexually transmitted infection, especially among high‐risk groups [11].

HAV transmission occurs mainly via the fecal‐oral route, through ingestion of water and food contaminated with feces from infected individuals or through direct person‐to‐person contact. These sources of infection are frequently reported during the occurrence of jaundice outbreaks. Food contamination includes mainly seafood (such as mussels and oysters), salads, fruits, and raw vegetables that may be contaminated with feces. The risk of direct person‐to‐person contamination increases with inadequate hygiene practices and sharing personal objects, especially when there are infected and susceptible individuals in the same environment, such as in domestic settings through recurrent intimate contact among family members, and in daycare centers and schools with the constant presence of children as reservoirs and sources of transmission to the population. Additionally, transmission can occur through sexual practices from direct contact with anatomical sites contaminated with feces, specifically through oral and anal sex practices. This route of contamination is directly influenced by individual lifestyle habits and is higher among MSM with multiple sexual partners and higher frequency of oroanal sex. Although HAV transmission via the parenteral route is rare, it can occur through blood transfusion or blood product derivatives from infected donors who donate during the viremia period. Transmission has also been reported among injectable drug users [4].

Few studies have explored the epidemiological and molecular aspects of HAV infection in patients with suspected acute hepatitis across various regions of a large country such as Brazil. This study aims to (1) assess HAV infection in suspected acute hepatitis patients at public healthcare institutions; (2) evaluate the proportion immunized against hepatitis; (3) identify HAV genotypes; (4) examine the association between HAV infection, demographic and clinical data, as well as exposure to risk factors. The findings from this study can help in developing targeted strategies for preventing and controlling HAV infection and aid in analyzing the evolutionary characteristics of the HAV genomes identified.

## 2 Materials and Methods

### 2.1 Patients and Samples

The study population consisted of individuals sequentially attended from October 2019 to May 2023 and were seeking care with clinical symptoms and signs suggestive of acute liver disease in a network of assistance to viral hepatitis, distributed throughout the five Brazilian regions: North, Northeast, South, Southeast, and Central West. They were sequentially attended from October 2019 to May 2023 and undergo tests for HAV (anti‐HAV IgG/IgM and PCR) from blood samples collected through the observational, prospective, and multicenter study entitled “Study of the epidemiological and clinical characteristics of acute viral hepatitis in Brazilian healthcare services.” [12]

The eligibility criteria were defined as: (1) Symptomatic jaundice: sudden appearance of jaundice within 6 months before the start of the study, with or without additional symptoms such as fever, malaise, nausea, vomiting, myalgia, acholic stools, and dark urine; in laboratory investigations, the patient had an increase in aminotransferase levels ≥ 1.5 times the upper limit of normal‐ ULN); (2) Symptomatic non‐jaundice: participants without jaundice but presenting with one or more symptoms such as fever, malaise, nausea, vomiting, myalgia; in laboratory investigations, they had an increase in aminotransferase levels ≥ 1.5 times the ULN); (3) Asymptomatic non‐jaundice: participants without jaundice or symptoms suggestive of acute hepatitis, but with a sudden increase in aminotransferase levels ≥ 1.5 times the ULN and/or participants with a known recognized close contact with a patient with confirmed viral hepatitis.

Blood samples collected by venipuncture, were properly identified with sequential labels provided and identified with the patients' full names and then centrifuged for serum or plasma separation. After collection, the samples were adequately processed, stored, and subsequently sent for analysis along with the completed documents to the Albert Einstein Clinical Pathology Laboratory in São Paulo, capital city of São Paulo State, Brazil.

This study was approved by the institutional research boards of all participating institutions (CONEP‐CAAE 00952818.4.1001.0071) and was previously described [12]. The inclusion criteria were individuals aged ≥ 18 years who signed the Informed Consent Form (ICF) or had their fingerprint collected in the ICF from illiterate patients. ICF was signed by a close relative if the patient was bedridden or unable to sign it. The study protocol conforms to the ethical guides of the 1975 Declaration of Helsinki reflected in a priori approval by the institution´s human research committee.

### 2.2 Epidemiological Data

A standardized questionnaire was used to collect demographic data (sex at birth, gender, age, and geographical origin), information on viral infection (anti‐HAV IgG and IgM, HAV RNA, HAV genotype through sequencing) and individual risk factors. Risk factors included questions on basic sanitation, contact with confirmed or suspected cases of viral hepatitis, close personal contacts (households, homeless people, or children in daycare centers), sexual behavior—sex at birth, sexual orientation, gender identity and number of partners, use of injectable medications or drugs etc.) Collected data were digitally completed through the REDCap electronic system, allowing for real‐time information storage and the generation of customized reports for data analysis. In addition, the participants' privacy and confidentiality were ensured through data encryption [13].

### 2.3 Laboratory Tests

#### 2.3.1 Serology of HAV

For the qualitative detection of IgM and IgG antibodies against HAV in human serum, all samples were analyzed using a chemiluminescent microparticle immunoassay (CMIA). These assays were performed using the HAVAb‐IgM and IgG Kit—Architect, Abbott Diagnostics, Abbott Park, IL, USA. For these serological tests, the procedures and interpretation of results were conducted according to the manufacturer's—https://www.abbott.com/for-healthcare-professionals/diagnostics.html.

#### 2.3.2 HAV RNA Detection and Genotyping

All samples underwent RNA viral nucleic acid extraction followed by HAV RNA detection through PCR and sequencing for genotyping.

##### 2.3.2.1 Purification of Viral RNA

RNA extraction from 350 µL of serum was carried out using the automated QIASymphony method, employing the QIAsymphony DSP Virus/Pathogen Mini Kit. (Qiagen, Hilden, Germany) The extracted RNA was eluted in 60 µL, following the manufacturer's instructions.

##### 2.3.2.2 Reverse Transcription—Polymerase Chain Reaction (RT‐PCR)

Subsequently, the extracted RNA underwent PCR amplification using a commercial real‐time PCR assay, RealStar HAV RT‐PCR 1.0 kit (Altona Diagnostics GmbH, Hamburg, Germany), considering samples with a Cycle Threshold (Ct) value ≤ 40 as positive for HAV.

##### 2.3.2.3 Genotyping

###### RT‐PCR and Nested PCR

As described previously [6], the samples underwent hemi‐nested PCR amplification using previously described primers. A SuperScript III One Step RT‐PCR System with Platinum Taq DNA Polymerase (Invitrogen, Carlsbad, CA, USA) was used for reverse transcription and the first round of amplification and the Platinum Taq DNA Polymerase enzyme (Invitrogen, Carlsbad, CA, USA) was used for the second round. The initial step for HAV RNA detection involved reverse transcription followed by a hemi‐nested PCR amplifying a 264‐base pair (bp) fragment covering the VP1/2A region of the HAV genome. Primers 2950F (5′‐TTGTCTGTCACAGAACAATCAG‐3′) and 3308R (5′‐AGTCACACCTTCCCAGGAAAACTT‐3′) were used for reverse transcription and the first round of amplification under the following thermocycling conditions: reverse transcription at 50°C for 30 min; Taq polymerase activation at 94°C for 2 min; and 35 cycles at 94°C for 15 s, 55°C for 30 s, and 68°C for 30 s. For the second round of this hemi‐nested PCR, primers 2950 F and 3217 R (5′‐AGGGGGTGGAAGTACTTCATTTGA3′) were used under the following thermocycling conditions: 94°C for 2 min and 35 cycles at 94°C for 15 s, 55°C for 30 s, and 72°C for 30 s.

###### Sanger Sequencing

The amplified fragments were purified using the ExoSAP‐IT PCR Cleanup Kit (Thermo Fisher, Waltham, MA, USA) and utilized as a template for sequencing with the BigDye Terminator Cycle Sequencing Kit version 3.1 (Thermo Fisher, Waltham, MA, USA) and an automated sequencer (3500 Genetic Analyzer, Thermo Fisher, Waltham, MA, USA). For genotype/subgenotype classification, the sequences of each region from all amplified samples were aligned using the ClustalW program integrated into BioEdit software version 7.0.8 [14]. For genotype determination, a phylogenetic analysis was conducted in MEGA X [15], using a maximum likelihood method with a Tamura three parameter model (data set with 264 bp VP1/2A sequences) as determined by the model selection analysis of MEGA X.

#### 2.3.3 Metagenomic Next Generation Sequencing (mNGS)

In parallel, another approach of total RNA shotgun metagenomics and computational analyses was applied to get complete or almost complete full‐length sequences of HAV genomes to identify possible viral transmission clusters from the positive samples originated from different states in Brazil. Total RNA isolation from plasma samples was carried out according to the manufacturer's protocol (QIAamp Viral RNA, QIAGEN, Hilden, Germany). Subsequently, RNA was purified and concentrated using the RNA Clean & Concentrator kit (Zymo Research, Irvine, CA, USA), including treatment with DNase I, followed by depletion of human ribosomal RNA using the QIAseq Fast Select RNA Removal kit (QIAGEN). Finally, the samples underwent random cDNA amplification following a methodology previously described with some modifications [16]. The amplified cDNA was used as input for mNGS library preparation with the Nextera XT DNA Sample Prep Kit. The resulting libraries were quantified, mixed in equimolar amounts, and subjected to 150 bp paired end reads using Illumina NextSeq. 550 sequencing systems (Illumina, San Diego, CA).

##### 2.3.3.1 Bioinformatics and Phylogenetics Analyses

Bioinformatics analysis of metagenomic sequencing data was performed using Varsmetagen (https://app.varsmetagen.com/), an on‐line platform for analyses of microbial NGS data. Shortly, Varsmetagen performs raw data quality control with fastqc and cutadapt, human decontamination through mapping to human genome (build HG38) using bwa aligner, pathogen identification with Kraken2 [17] and a customized database of RefSeq NCBI complete genomes and finding confirmation through mapping and calculating quality metrics to specific target genomes. Complete HAV genomes were assembled using two techniques: consensus generation using target reference mapping, and de novo assembly with SPAdes [18]. Consensus and contigs sequences were manually curated to recover a complete genome, whenever possible. HAV genomes recovered in this study were aligned along with representative HAV genomes from different genotypes and geographic regions as available in the NCBI library, using MAFFT (Multiple Alignment using Fast Fourier Transform)—(https://mafft.cbrc.jp). Multiple sequence alignment was inspected to identify possibly artefacts and then submitted to maximum likelihood phylogenetic trees using RAxML [19] with GTR + G + I and 100 bootstraps replicas. Complete genomes were submitted to Genbank under bioproject PRJNA1064948.

### 2.4 Statistical Procedures

The initial step was to compare the frequency of the different variables in two different analysis comparing (1) patients immunized against HAV [anti‐HAV IgG (+)] vs. those not immunized [anti‐HAV IgG (−)] and (2) patients with acute hepatitis A (AHA) [anti‐HAV IgM or HAV RNA (+)] vs. those without AHA [anti‐HAV IgM and HAV RNA (−)]. The data were analyzed using Student's *T*‐test and the *χ* ^2^ test for continuous quantitative variables and categorical variables, respectively. The significance level was set at 5%.

A backward stepwise logistic regression model was conducted; variables with *p*‐values < 0.05 from the bivariate analyses were used as predictors for the two outcomes, namely: presence of anti‐HAV IgG antibodies; presence of anti‐HAV IgM antibodies or HAV RNA. Odds ratios (OR) with CIs of 95% were calculated. Statistical analyses were performed using the RStudio statistical software [20].

## 3 Results

### 3.1 Analysis of Epidemiological, Clinical and Laboratorial Data

From October 2019 to May 2023, a total of 1752 samples were collected, of which 1727 were considered valid according to the protocols established by the study. These samples came from 31 reference centers distributed across 16 states covering the five regions of Brazil. Out of these 1727 samples, six did not have sufficient volume to undergo serological testing for Anti‐HAV IgG. Therefore, among 1721 samples subjected to the test, 78.5% (*n* = 1352) tested positive for Anti‐HAV IgG.

**Figure 1:**
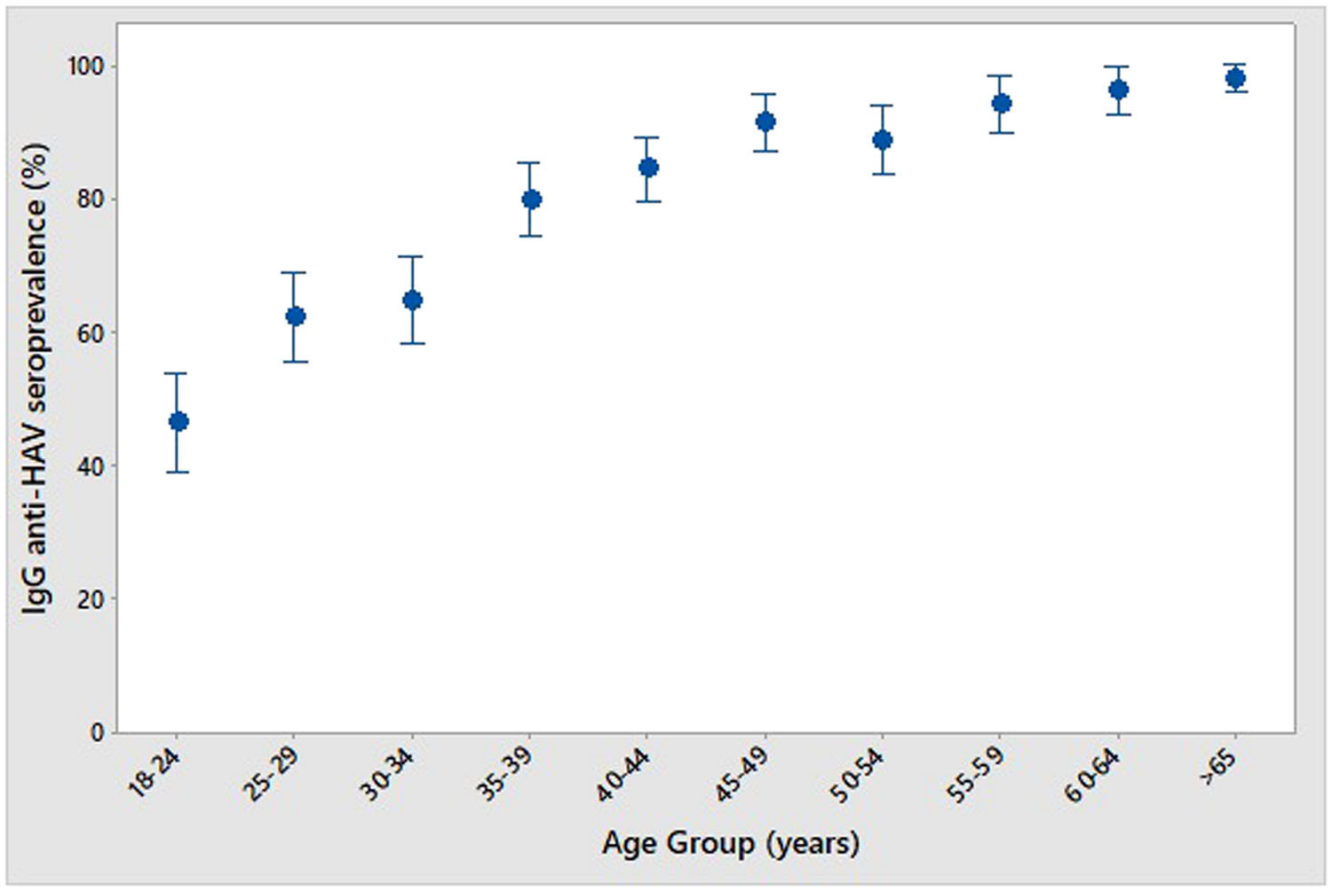
IgG anti‐HAV seroprevalence by age group.

Table 1 shows the demographic and behavioral profile of patients statistically associated with Anti‐HAV IgG (+): older patients (*p* < 0.001); males (*p* = 0.013); from the North region (*p* < 0.001); from rural areas (*p* = 0.007); heterosexuals (*p* = 0.010); cisgender men (*p* = 0.039); afro descendants (*p* < 0.001); married or in common law relationship (*p* = 0.045); individuals without access to sewage and treated water (*p* < 0.001); without raw or undercooked seafood consummation (*p* < 0.001); without sexual partners in the last 6 months (*p* < 0.001*)*. The use of injectable drugs was not related to anti‐HAV IgG (*p* = 0.6). Figure 1 shows the age distribution of anti‐HAV IgG. It is shown that from 18 to 24 years, about half of the individuals were IgG (+), what increased to more than 60% from 25 to 34 years old; after that age range, IgG (+) levels increased from more than 80% to more than 95% after 55 years old.

**Table 1:** Demographic and exposure to risk factors in patients with anti‐HAV IgG (−) and (+).

	Total	Number of cases of Anti‐ HAV IgG (−)	Number of cases of Anti‐ HAV IgG (+)	*p* value^a^
	N = 1721	N = 369 (%)	N = 1352 (%)	
Age^b^	41 (31, 53)	30 (24, 37)	44 (34, 56)	***< *** **0.001**
Sex at birth				**0.013**
Female	730	178 (24%)	552 (76%)	
Male	991	191 (19%)	800 (81%)	
Region of residence				**< 0.001**
Midwest	125	27 (22%)	98 (78%)	
Northeast	211	40 (19%)	171 (81%)	
North	244	20 (8%)	224 (92%)	
Southeast	755	178 (24%)	577 (76%)	
South	386	104 (27%)	282 (73%)	
Geographical characteristics of the place of residence				**0.007**
Rural area	123	14 (11%)	109 (89%)	
Urban area	1595	353 (22%)	1242 (78%)	
Missing	3	2	1	
Sexual orientation				**0.010**
Heterosexual	1396	281 (20%)	1115 (80%)	
Nonheterosexual	246	68 (28%)	178 (72%)	
Missing	79	20	59	
Gender identity				**0.039**
Cis man	943	181 (19%)	762 (81%)	
Trans man	1	1 (100%)	0 (0%)	
Cis woman	705	169 (24%)	536 (76%)	
Trans woman	9	2 (22%)	7 (78%)	
Nonbinary	4	1 (25%)	3 (75%)	
Missing	59	15	44	
Race/color				**< 0.001**
Afro descendant	479	82 (17%)	397 (83%)	
Non‐afro descendant	723	192 (27%)	531 (73%)	
Unknown	519	95	424	
Marital status				**0.045**
Married/Common‐law relationship	783	150 (19%)	633 (81%)	
Divorced/Single/Separated/Widowed	906	211 (23%)	695 (77%)	
Missing	32	8	24	
Access to sewage and treated water				**< 0.001**
No	263	27 (10%)	236 (90%)	
Yes	1456	341 (23%)	1115 (77%)	
Missing	2	1	1	
Did you consume raw or undercooked seafood?				**< 0.001**
No	1351	256 (19%)	1095 (81%)	
Yes	366	111 (30%)	255 (70%)	
Missing	4	2	2	
In the last 6 months, how many people have you had sex with?				**< 0.001**
No partner	350	47 (13%)	303 (87%)	
1 partner	1074	239 (22%)	835 (78%)	
2 or more partners	281	76 (27%)	205 (73%)	
Missing	16	7	9	
Have you injected drugs?				0.6
No	1663	358 (22%)	1305 (78%)	
Yes, more than 6 months ago	24	3 (13%)	21 (88%)	
Yes, less than 6 months ago	18	4 (22%)	14 (78%)	
Missing	16	4	12	

*Note:* Bold values are statistically significant.

^a^ Age ‐ Wilcoxon test; Sex, region, geographical caracteristic, sexual orientation, race, marital status, sewage, seafood, sex 6 months, inclusion criteria ‐ Chi‐square test; Gender, inject drug ‐ Fisher exact test.

^b^ Age is expressed as median (interquartile range IQR 25%; 75%). All the other variables are expressed as number (percentage).

Table 2 shows the results of logistic regression analysis for Anti‐HAV IgG seropositivity. Advanced age was significantly associated with seropositivity (OR = 1.09; 95% CI: 1.07–1.10; *p* < 0.001). Males had higher probability of seropositivity compared to females (OR = 1.51; 95% CI: 1.15–1.99; *p* = 0.003). Among the regions of residence, the North region showed a significant increase in the odds of seropositivity compared to all the other four regions analyzed. Access to sewage and treated water was associated with a significant reduction in the odds of seropositivity (OR = 0.51; 95% CI: 0.31–0.83; *p* = 0.008). Patients with one sexual partner were associated with a higher probability of seropositivity (*p* = 0.011) when compared to those who had no partner or more than 1 partner.

**Table 2:** Logistic regression analysis for factors associated with anti‐HAV IgG seropositivity.

Characteristic	OR^a^	95% CI^a^	*p* value
Age	1.09	1.07, 1.10	**< 0.001**
Sex			
Female	—	—	
Male	1.51	1.15, 1.99	**0.003**
Region			
North	—	—	
Midwest	0.43	0.21, 0.86	**0.018**
Northeast	0.44	0.23, 0.81	**0.009**
Southeast	0.31	0.17, 0.52	**< 0.001**
South	0.22	0.12, 0.38	**< 0.001**
Sewage water			
No	—	—	
Yes	0.51	0.31, 0.83	**0.008**
Number of sexual partners			
No partner	—	—	
1 partner	0.61	0.41, 0.89	**0.011**
2 or more partners	0.73	0.46, 1.16	0.2

*Note:* Bold values are statistically significant.

^a^ CI = confidence interval, OR = odds ratio.

Of the 1721 samples, 108 (6.3%) were defined as acute hepatitis A (AHA): 99 IgM (+) [96 PCR (+) and 3 PCR (−)] and 9 PCR (+) (all of them IgM negative). Table 3 displays the demographic and behavioral profile of patients with AHA: more frequent in younger patients (*p* < 0.001), males (*p* < 0.001), from the South and Southeast regions (*p* < 0.001), from urban areas (*p* = 0.005), non‐heterosexuals (*p* < 0.001), cisgender men/transgender women/nonbinary (*p* < 0.001), divorced/single/separated/widowed (*p* = 0.032); with access to sewage and treated water (*p* = 0.038); in those that have consumed raw seafood (*p* < 0.001*);* with 2 or more sexual patterns in the last 6 months (*p* < 0.001*)*. Race and use of injectable drugs were not related to AHA (*p* = 0.2 and *p* = 0.6, respectively). AHA participants had more frequently symptomatic jaundice clinical pictures (*p* < 0.001).

**Table 3:** Demographic and behavioral profile comparing patients with and without acute hepatitis A (AHA) [anti‐HAV IgM or HAV RNA (+)] vs. those without AHA [anti‐HAV IgM and HAV RNA (−), respectively].

	Total	Number of cases without AHA	Number of cases with AHA	*p* value^a^
	*N* = 1727	*N* = 1619	*N* = 108	
Age^b^	41 (30, 53)	41 (31, 54)	34 (28, 40)	**< 0.001**
Sex at birth				**< 0.001**
Female	732	710 (97%)	22 (3%)	
Male	995	909 (91%)	86 (9%)	
Region of residence				**< 0.001**
Midwest	125	124 (99%)	1(1%)	
Northeast	212	211 (100%)	1 (0%)	
North	246	245 (100%)	1 (0%)	
Southeast	757	690 (91%)	67 (9%)	
South	387	349 (90%)	38 (10%)	
Geographical characteristics of the place of residence				**0.005**
Rural area	124	124 (100%)	0 (0%)	
Urban area	1600	1492 (93%)	108 (7%)	
Missing	3	3		
Sexual orientation				**< 0.001**
Heterosexual	1402	1346 (96%)	56 (4%)	
Nonheterosexual	246	204 (83%)	42 (17%)	
Missing	79	69	10	
Gender identity				**< 0.001**
Cis man	947	872 (92%)	75 (8%)	
Trans man	1	1 (100%)	0 (0%)	
Cis woman	707	685 (97%)	22 (3%)	
Trans woman	9	8 (89%)	1 (11%)	
Nonbinary	4	3 (75%)	1 (25%)	
Missing	59	50	9	
Race/color				0.2
Afro descendants	480	452 (94%)	28 (6%)	
Non–afro descendants	724	668 (92%)	56 (8%)	
Unknown	523	499	24	
Marital status				**0.032**
Married/Common‐law relationship	786	748 (95%)	38 (5%)	
Divorced/Single/Separated/Widowed	909	841 (93%)	68 (7%)	
Missing	32	30	2	
Access to sewage and treated water				0.038
No	264	255 (97%)	9 (3%)	
Yes	1461	1362 (93%)	99 (7%)	
Missing	2	2		
Did you consume raw or undercooked seafood?				**< 0.001**
No	1355	1296 (96%)	59 (4%)	
Yes	368	319 (87%)	49 (13%)	
Missing	4	4		
In the last 6 months, how many people have you had sex with?				**< 0.001**
No partner	352	346 (98%)	6 (2%)	
1 partner	1077	1018 (95%)	59 (5%)	
2 or more partners	282	239 (85%)	43 (15%)	
Missing	16	16		
Have you injected drugs?				0.6
No	1669	1563 (94%)	106 (6%)	
Yes, more than 6 months ago	24	24 (100%)	0 (0%)	
Yes, less than 6 months ago	18	17 (94%)	1 (6%)	
Missing	16	15	1	
Inclusion Criteria				**< 0.001**
Asymptomatic anicteric	241	236 (98%)	5 (2%)	
Anicteric symptomatic	643	633 (98%)	10 (2%)	
Symptomatic jaundice	842	749 (89%)	93 (11%)	
Missing	1	1		

*Note:* Bold values are statistically significant.

^a^ Age ‐ Wilcoxon test; Sex, region, geographical caracteristic, sexual orientation, race, marital status, sewage, seafood, sex 6 months, inclusion criteria ‐ Chi‐square test; Gender, inject drug ‐ Fisher exact test.

^b^ Age is expressed as median (interquartile range IQR 25%; 75%). All the other variables are expressed as number (percentage).

**Figure 2:**
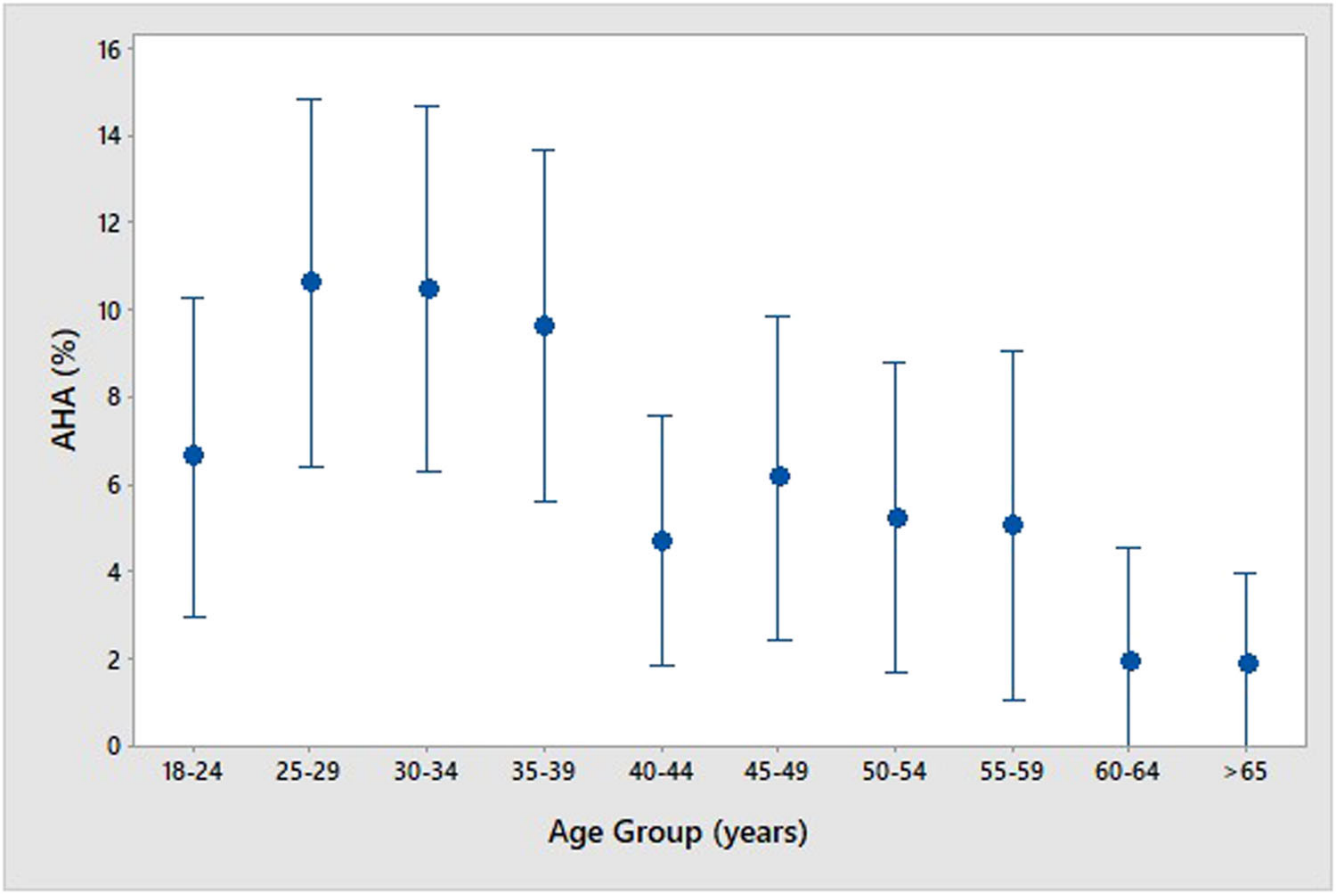
Acute hepatitis A (AHA) [anti‐HAV IgM or HAV RNA (+)] prevalence by age group.

Figure 2 shows the age distribution of AHA. It is shown that from 25 to 39 years old, we found the highest percentages of AHA (around 10%) and that in the other age groups, it is always less than 7%, going down to around 2% after 60 years old.

Regarding the clinical symptoms of patients diagnosed with acute hepatitis A, 83% presented dark urine, 87% jaundice, 78% nausea, 74% anorexia, 62% fever, 56% clear stools, 46% vomiting, 25% hepatomegaly, 15% rash, 14% splenomegaly, and 8% lymphadenopathy. The mean total serum bilirubin level for those diagnosed with acute hepatitis A was 6.54 mg/dL, with direct bilirubin at 5.10 mg/dL and indirect bilirubin at 0.97 mg/dL. The average levels of AST and ALT were 1150 U/L and 598.70 U/L, respectively.

Table 4 shows the results of logistic regression analysis for AHA. Age was inversely associated with seropositivity (OR = 0.96; 95% CI: 0.95 to 0.98; *p* < 0.001). Males had a 2.24 times higher probability of seropositivity compared to females (OR = 2.24; 95% CI: 1.36 to 3.83; *p* = 0.002). The North region showed a significant reduction in the odds of AHA compared to the South region (OR = 0.04; 95% CI: 0.00 to 0.17; *p* = 0.001), as did the Midwest region (OR = 0.09; 95% CI: 0.00 to 0.42; *p* = 0.017), and the Northeast region (OR = 0.05; 95% CI: 0.00 to 0.25; *p* = 0.004), but it was not different from the Southeast region (OR = 0.82; 95% CI: 0.53 to 1.29, *p* = 0.4). Consumption of seafood was significantly associated with increased odds of seropositivity (OR = 1.93; 95% CI: 1.25 to 2.96; *p* = 0.003). Patients with 1 and 2 or more sexual partners were associated with a higher likelihood of AHA (OR = 2.99, 95% CI: 1.36 to 7.90, *p* = 0.013 and OR = 4.42; 95% CI: 1.91 to 12.1; *p* = 0.001, respectively) compared to those with no partners.

**Table 4:** Logistic regression analysis for factors associated with acute hepatitis A (AHA) [anti‐HAV IgM or HAV RNA (+)].

Characteristic	OR^a^	95% CI^a^	*p* value
Age	0.96	0.95, 0.98	**< 0.001**
Sex			
Female	—	—	
Male	2.24	1.36, 3.83	**0.002**
Region			
South	—	—	
North	0.04	0.00, 0.17	**0.001**
Midwest	0.09	0.00, 0.42	**0.017**
Northeast	0.05	0.00, 0.25	**0.004**
Southeast	0.82	0.53, 1.29	0.4
Seafood			
No	—	—	
Yes	1.93	1.25, 2.96	**0.003**
Number of partners			
No partner	—	—	
1 partner	2.99	1.36, 7.90	**0.013**
2 or more partners	4.42	1.91, 12.1	**0.001**

*Note:* Bold values are statistically significant.

^a^ CI = confidence interval, OR = odds ratio.

### 3.2 Genotypes of HAV Determined by Sanger Sequencing and Metagenomics Next Generation Sequencing (mNGS)

Out of 105 cases positive for viral RNA, 92 were sequenced for genotyping: 16 by Sanger, 50 by both Sanger and mNGS, and 26 by mNGS. Altogether, all the samples from the different Brazilian regions harbored genotype I.A.

### 3.3 Complete HAV Genomes Recovered From mNGS

**Figure 3:**
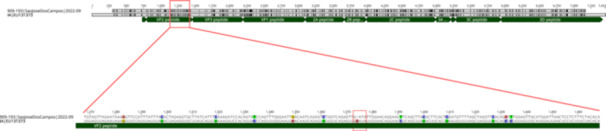
Whole genome alignment of the HAV sequence from São José dos Campos collected in September 2022 against reference strain EU131373. The alignment reveals a 1‐nucleotide deletion within the VP2 protein‐coding region. A detailed view of the alignment highlights the specific location of the deletion, which may have implications for protein function, viral fitness or immunogenicity.

In 76 samples, mNGS detected at least one fragment corresponding to HAV RNA and in 35 of them, almost full‐length genomes were successfully sequenced. The length of the genomes ranged from 6625 to 7462 bp, with an average GC content of 38%, and they exhibited 89%–100% completeness. Pairwise nucleotide alignments classified these genomes as genotype I.A, and their identities ranged from 94.7% to 99.7%. Despite high identities, and that all genomes belong to genotype I.A, it is remarkable that genomes recovered from HAV patients analyzed in this study showed a reasonable degree of novelty when compared with HAV genomes from other geographic regions. These results suggest an unexplored diversity in the HAV evolutionary landscape. Approximately 450 mutations, corresponding to up to 6% of the differences among genomes, were identified, most of them found in the structural proteins VP1, VP2, and VP3. It is noteworthy the finding a deletion never reported before in the capsid protein and VP2 antigen of the hepatitis A virus (Figure 3).

### 3.4 Phylogenetic Analyses

The phylogenetic analysis of 264‐ bp from VP1/2A region of the HAV genome sequenced by Sanger methodology from 66 samples shows that all of them were classified as subgenotype I.A. and most (83.3%; 55/66) were close related with reference clusters (VRD_521_2016 and RIM‐HAV16‐090) of HAV sequences identified in outbreaks that occurred between 2016 and 2018 in some European countries affected unvaccinated young adult men, particularly men who have sex with men (MSM). Other Brazilian sequences isolated from AHA that occurred in São Paulo (MG049743) and in Rio de Janeiro (MK170459, MK170458) in 2017 (the beginning of MSM HAV outbreak in these cities) also grouped with reference cluster VRD_521_2016. Strains related to VRD_521_2016 cluster were the most widespread in the different Brazilian cities where cases of AHA were identified in this study (Figure S1).

**Figure 4:**
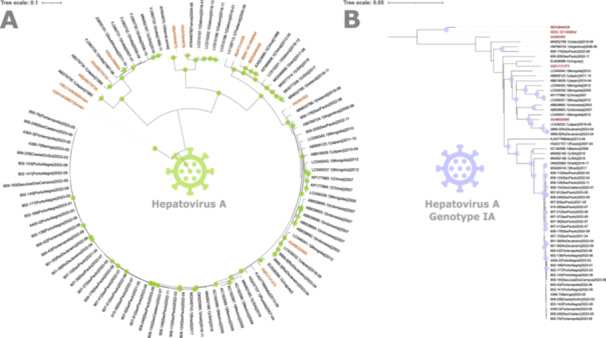
Rooted phylogenetic trees based on complete genomes of the hepatitis A virus reconstructed using maximum likelihood. Names in red are the samples with reference genotype. (A) Phylogenetic tree with all genotypes of HAV; (B) Phylogenetic tree only with genotype IA genomes plus IB external groups. Branches with bootstrap support above 70% are marked with green (A) or purple (B) circles next to the corresponding branch.

To analyze the evolutionary relationship between the 35 almost full‐length HAV genomes recovered in this study two phylogenetic reconstructions were performed. The first phylogenetic tree contains all genotypes of HAV, along with external groups and shows that all genomes recovered in this study were correctly placed along with public genotypes I.A (Figure 4A). The second phylogenetic tree contain only sequences of genotype I to analyzing in detail the relationship among the HAV I.A Brazilian sequences characterized in this study and sequences from other countries. Two main subgroups were identified among genotypes I.A: A major group of Brazilian sequences with cosmopolitan sequences from North America, South America and Europe; and a second minor group of Brazilian sequences with sequences of Asia and South America (Figure 4B).

## 4 Discussion

This multicenter study conducted in public health services in Brazil evaluated the prevalence of anti‐HAV IgG and IgM antibodies and HAV RNA for hepatitis A in individuals with clinical and/or laboratory suspicion of acute hepatitis. Anti‐HAV IgG showed an overall prevalence of 78.6%, ranging from 74% in the Southern region to 91% in the Northern region of the country (*p* < 0.001). Similar data have also been found in other studies showing the Northern region of the country with the highest prevalence and the Southern region with the lowest prevalence [21]. This fact is likely related to basic sanitation conditions, such as access to treated water and sewage characteristics of each region. Strengthening this data, a direct relationship between seropositivity for anti‐HAV IgG and access to sewage and treated water was found, as also demonstrated in other studies [22]. Considering the transmission routes of hepatitis A, the provision of safe drinking water and the elimination of inadequate sewage in communities, together with personal hygiene practices such as regular hand washing, reduce the spread of HAV [22]. Brazil has been considered a country of intermediate prevalence and in recent studies seems to be in transition, being classified as low‐intermediate [21].

The average age was higher in individuals with positive anti‐HAV IgG, 45 years versus 31 years (*p* < 0.001). This characteristic is well described in the literature, and several studies show that the prevalence of anti‐HAV IgG increases with age, even in countries with low prevalence [23]. A previous study in Latin America evaluated four different regions of Brazil and described an overall prevalence of 64.7% of anti‐HAV IgG, reaching 95% in individuals between 31 and 41 years old, similar to data found in Chile and Mexico [24]. In another study, 11 177 individuals in Korea (between 2010 and 2014) were tested for anti‐HAV IgG. The age group of 20 to 24 years had a positivity rate of 12.7%, from 35 to 39 years old, 50.5%, with 90% of the population above 45 years old showing positive anti‐HAV IgG [25]. Controversial data were found in blood donors in the United States of America. The overall prevalence of anti‐HAV IgG was 60%, however, when analyzing age groups, a prevalence of 67% was found in individuals aged 16 to 19 years, decreasing to 54% between 40 and 49 years old, increasing again to 70% between 80 and 93 years old. This overall high background rates of IgG anti‐HAV in the general blood donor population in the USA particularly in younger age is presumably due to vaccinated individuals [26].

The prevalence of individuals with AHA was 6.3%. It was observed that the lower the seroprevalence of anti‐HAV IgG (+) in the region, the higher the chances of acute infection, including symptomatic jaundice. The Southeast and South regions showed lower prevalences of anti‐HAV IgG (+), and the highest incidence of anti‐HAV IgM (+) and RNA HAV (+). This data is consistent with previous studies showing that regions with better sanitation conditions have more adults susceptible to HAV infection, with these infections being more symptomatic and thus with a higher number of notifications [27].

In the present study, nearly 80% of HAV cases were found in young men, with the Southern and Southeastern regions concentrating 97% of these cases. The increase in hepatitis A incidence among young men was described in some regions with low endemicity of Brazil [6]. Additional, other risk factors were identified for HAV, including living in urban areas, nonheterosexual orientation, having two or more sexual partners in the last 6 months, and consumption of raw or undercooked seafood. Some of these risk factors have already been well described in the literature, including their association with outbreaks in specific populations [28]. Therefore, it is of fundamental importance to discuss the implementation of hepatitis A vaccination, especially in high‐risk populations such as young men living in urban areas of the Southern and Southeastern regions of Brazil.

Individuals with HAV were highly symptomatic. The main symptoms found were jaundice (87%), dark urine (83%), nausea (78%), anorexia (74%), fever (62%), pale stools (56%), and vomiting (46%). It is well described in the literature that the risk of clinical illness after HAV infection is directly related to the age of the infected person. When infection occurs during early childhood, it is mostly asymptomatic. Therefore, although adults exposed to HAV may not show symptoms, there is a higher probability of developing a severe icteric illness requiring hospitalization and developing complications such as fulminant hepatitis [29]. Previous studies with HAV in adults have shown that despite a low fatality rate, this risk appears to increase with age [30].

This study contributes new and comprehensive data to the scientific literature based on its molecular and phylogenetic findings. While HAV genotype prevalence in Brazil has not been extensively examined in prior studies, a few investigations have reported hepatitis virus molecular and serological prevalence only in specific communities [6, 31]. The Genotype I.A was the only one identified among AHA cases in Brazil detected by our study. HAV genotype I.A changed during its local evolution: 450 mutations found (up to 6% of the differences among genomes) were mostly found in structural proteins VP1, VP2, and VP3, implicating in possible changes in the immunoreactivity of these agents. Additionally, such changes could impact vaccine efficacy and contribute to the viral evolutionary dynamics [32].

A novel deletion in VP2 capsid protein has been identified in one case that can lead to reduced viral stability and infectivity, potentially impairing its ability to attach to host cell, but it may also affect the immune response and alter the viral pathogenicity. Mutations in key virulence genes can impact the virus's fitness, replication, and antigenicity. VP2 interacts with the ESCRT (Endosomal Sorting Complex Required for Transport) machinery through YPX₃L motifs in its C‐terminal region, which recruit PDCD6IP (Programmed Cell Death 6‐Interacting Protein) to help with the virus's quasi‐envelopment and egress [33].

The phylogeny of the sequences characterized in this study point to the wide dissemination of strains that were introduced into Brazil in 2016–2017 when begging MSM outbreak in our country. Previous studies that analyzed AHA cases identified during these outbreaks in São Paulo and in Rio de Janeiro shows that most of the strains identified were closely related to the strains identified in the outbreaks in different European countries during the period from 2016 to 2018 (VRD_521_2016, RIMV‐HAV16‐090 and V16‐25801).

Nevertheless, the complete genome phylogenetic analyses allowed for a more refined understanding of differences and evolutionary paths, leading to the clear identification of distinct groups of the genotype I.A. Some of these groups may be indicative of recent introductions or reintroduction of the virus from foreign cases, as shown in Figure 4. Alternatively, they may reflect the transfer of the virus among Brazil and other Latin America countries (e.g., Haiti, Cuba and Venezuela), as an important migration was recently reported from these countries (https://www.unhcr.org/countries/brazil). Regardless, further investigation is warranted for a more comprehensive understanding. This study has significantly advanced our understanding of HAV evolution and the fact that the complete genomes have been made publicly available will undoubtedly facilitate further investigations in this area.

As a limitation, individuals under the age of 18 were not included in the study, which may restrict the evaluation of anti‐HAV IgG prevalence in the population and consequently the classification into low, intermediate, and high categories. This exclusion could potentially affect the generalizability of the findings, as hepatitis A infection patterns and immunity status can vary significantly across different age groups. Including individuals under 18 would have provided a more comprehensive understanding of HAV prevalence and immune status across the entire population. Future studies should consider including a wider age range to ensure more representative results and better inform public health interventions related to hepatitis A.

In conclusion: (1) The study confirmed the occurrence of HAV infection among individuals presenting with symptoms of acute hepatitis at public healthcare institutions. A notable proportion of these patients tested positive for anti‐HAV IgM antibodies, indicating recent or ongoing HAV infection and highlighting the continued circulation of HAV in the region; (2) Serological analysis revealed that a significant portion of the study population lacked total anti‐HAV antibodies, indicating susceptibility to HAV infection. This suggests gaps in immunization coverage or natural immunity, underscoring the need for enhanced hepatitis A vaccination strategies in the affected population; (3) Molecular characterization identified the circulation of HAV genotype I.A, consistent with patterns observed in Brazil and other parts of South America. This finding provides valuable epidemiological data and supports the importance of continuous molecular surveillance to detect shifts in circulating genotypes or emerging variants; (4) Statistical analysis demonstrated that AHA was more frequently diagnosed in nonheterosexual men with multiple sexual partners, particularly from urban areas in the South and Southeast regions of Brazil. Additionally, risk factors such as poor sanitation and limited access to clean water were linked to increased infection rates, reinforcing the need for targeted public health interventions addressing both behavioral and environmental vulnerabilities.

## Author Contributions

All authors have contributed according to the ICMJE definition of authorship: (1) substantial contributions to conception and design, acquisition of data, or analysis and interpretation of data; (2) drafting the article or revising it critically for important intellectual content; and (3) final approval of the version to be published.

## Ethics Statement

This study was approved by the institutional research boards of all participating institutions (CONEP‐CAAE 00952818.4.1001.0071).

## Consent

All patients signed the Informed Consent Form (ICF) or had their fingerprint collected in the ICF from illiterate patients. ICF was signed by a close relative if the patient was bedridden or unable to sign it.

## Conflicts of Interest

The authors declare no conflicts of interest.

## Supporting information


**SUPPLEMENTAL Figure 1 ‐ Phylogenetic analysis performed in MEGA X [15] utilized a maximum likelihood approach with a Tamura 3 parameter model (dataset comprising 264 bp VP1/2A sequences), as identified by the model selection analysis of MEGA X from the VP1/2A region of the HAV genome sequenced using Sanger methodology from 66 samples in this study, along with several samples from earlier studies that represent various genotypes**.

## Data Availability

The data that support the findings of this study are available on request from the corresponding author. The data are not publicly available due to privacy or ethical restrictions.
