# Supplementary figures and images for: A Comprehensive Molecular and Serological Investigation of Hepatitis A Virus Among Patients With Suspected Acute Hepatitis: A Brazilian Study
Source: J Med Virol. 2025 Jun 18;97(6):e70449. doi: 10.1002/jmv.70449 (PMC12175488; doi:10.1002/jmv.70449)

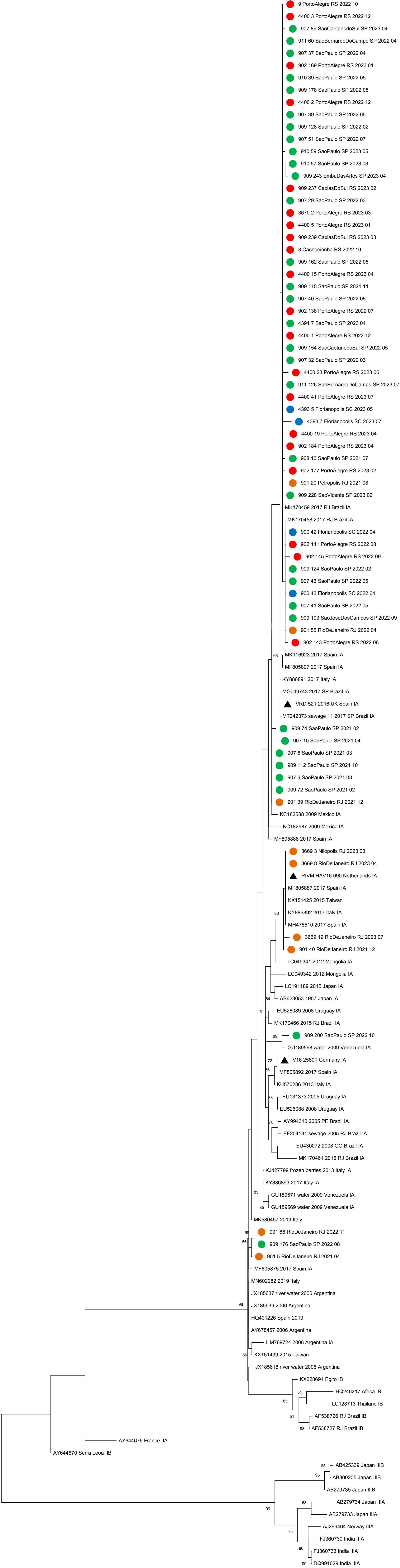

0.10

Supplement: Supplementary file 1 — SUPPLEMENTAL Figure 1 ‐ Phylogenetic analysis performed in MEGA X [15] utilized a maximum likelihood approach with a Tamura 3 parameter model (dataset comprising 264 bp VP1/2A sequences), as identified by the model selection analysis of MEGA X from the VP1/2A region of the HAV genome sequenced using Sanger methodology from 66 samples in this study, along with several samples from earlier studies that represent various genotypes. [file JMV-97-e70449-s001.pdf]
